# Empowering Aging in Place Through Community Health Innovation for Asian Older Adults

**DOI:** 10.1093/geroni/igaf122.751

**Published:** 2025-12-31

**Authors:** Bada Kang, Noriko Yamamoto-Mitani, Eleanor McConnell

## Abstract

With the super-aging of South Korea and Japan, enabling older adults to age in place has become a critical priority in both countries. This symposium explores transformative approaches that leverage digital technologies and community-driven initiatives in these countries to develop scalable solutions for aging in place. The first presentation introduces a university-led community health program in Japan, designed to establish a sustainable care-delivery system. This program focuses on care-competency education, healthy living environments, and mutual support among care professionals. Using a community-based participatory research approach, it seeks to extend healthy life expectancy by actively involving citizens in their own care. The second presentation highlights an educational program aimed at enhancing end-of-life care skills for both nurses and caregivers. The program creates an engaging and collaborative learning environment for palliative care by integrating dramatic e-learning, expert-led lectures, and professional shadowing. The third presentation examines caregiver burden in dementia care using multimodal monitoring techniques, including sweat patches, actigraphy, and surveys. In identifying the biological, psychological, and social factors contributing to caregiver burden, this study emphasizes the need for tailored interventions to improve care outcomes. The final presentation investigates the feasibility of mobile-based Ecological Momentary Assessment (EMA) for measuring social connectedness among older adults. Using real-time data collected from a senior living lab cohort, this study refines assessment methodologies to better understand social connectedness, emotions, and contextual influences. This symposium provides valuable insights into innovative solutions for aging in place to inform the future direction of community-based healthcare for older adults.

